# Aluminium Exposure in Understory Birds of Atlantic Forest Fragments in Brazil

**DOI:** 10.1007/s00128-026-04239-6

**Published:** 2026-04-17

**Authors:** Renata Constant de Amorim Lemos, Daniela Santos Anunciação, Gisele Maria Nunes Vieira, Márcio Amorim Efe

**Affiliations:** 1 Institute of Biological and Health Sciences, Postgraduate Program in Biological Diversity and Conservation in the Tropics, Federal University of Alagoas, Maceió, Alagoas Brazil; 2 Laboratory of Bioecology and Conservation of Neotropical Birds, Maceió, Brazil; 3 Laboratory of Instrumentation and Development in Analytical Chemistry, Maceió, Brazil

**Keywords:** Pernambuco Center of Endemism, Contaminants, Heavy metals, Threatened birds, Endemic birds

## Abstract

The combination of contaminated and fragmented habitat puts the most sensitive species at risk and can function as an ecological trap. Therefore, it is expected that forest birds that inhabit fragments surrounded by agricultural activities present some level of contamination by non-essential metals. Thus, this work sought to identify, using feathers, whether wild understory birds are contaminated by aluminum from sugarcane crops and agricultural. Thus, this work assessed body condition and sought to identify, using feathers, whether wild understory birds are contaminated by aluminum in forest fragments surrounded from sugarcane crops and agricultural pastures. The study was carried out, between September 2021 and October 2022, in three fragments of Atlantic Forest protected, in Alagoas, Brazil. For analysis, 10 breast feathers of 35 individuals, belonging to 15 species were collected and grouped by date and location. The samples were macerated, lyophilized, weighed, stored in a desiccator, and digested in a microwave oven, and analyzed using Optical Emission Spectrometry and inductively coupled plasma, to determine the concentrations of aluminum. The presence of aluminum was detected in all samples (57, 92—552, 62 mg/kg). There is no consensus on the exact universal cutoff value for aluminum contamination in feathers. However, values above 200 mg/kg are not considered normal. Furthermore, the results showed the need to pay attention in the protected areas studied, which may be functioning as ecological traps, bringing harm to the reproductive success of several species, especially endemic and threatened ones, which tend to have their populations already reduced for other reasons.

**Supplementary Information:**

The online version contains supplementary material available at 10.1007/s00128-026-04239-6.

## Introduction

The Atlantic Forest is considered a global hotspot (Myers et al. 2000) with a high level of biodiversity, endemism and threat (Tabarelli et al. 2004, 2006). In particular, the Atlantic Forest north of the São Francisco River, known as the Pernambuco Center of Endemism (Brown 1982; Prance 1982; Silva and Casteleti 2003, Peres et al. 2020) and considered one of the most important areas for bird conservation in Brazil as it is home to a large number of species threatened with extinction (Brooks and Ryland 2003; Silveira et al. 2003; IUCN 2024). Currently, is represented by small patches of forest (fragments) immersed in urban and agricultural matrices caused by the expansion of livestock and sugar cane farming (Coimbra-Filho and Câmara 1996), where conservation efforts are most urgent (Dias et al. 2023). In the sugar and alcohol production process, it is necessary to use fertilizers that may contain metals in their composition and contaminate environments and their fauna (Kojadinovic et al. 2007). Among these non-essential metals, aluminum (Al) is one of the most dangerous (Filizola et al. 2006). Generally, aluminum does not occur naturally in its metallic form, there is always a combination with other elements, mainly oxygen, forming an extremely hard oxide, alumina (Constantino et al. 2002).

The concentration of aluminum considered toxic to birds is not yet clearly defined in the scientific literature, but studies show that the measured concentrations are below 200 mg/kg (Shafaeipour et al. 2024, Elliott 2005). However, aluminum contamination can cause serious damage to human health (Behra et al. 2025) and to birds (Kojadinovic et al. 2007; Marchesi 2013; Innangi et al. 2019), which have specific eating habits, the ability to move, great sensitivity and low levels of detoxifying enzymes (Grue et al. 1984; Parker et al. 2000). Exposure of birds to these metals can affect their physiology, behavior, ability to resist disease and high mutation rates (Bickham et al. 2000). Generally, is possible to assess the level of this contamination through plumage in this group (Irena et al. 2017; Kitowski et al. 2017)since during the molting process, metals present in the blood accumulate in the feather (Hofer et al. 2010). Furthermore, birds are frequently used to assess environmental metal contamination** (**Berglund 2018) and feathers are a useful biomonitoring tool (Dauwe et al. 2003; Cardiel et al. 2011; Borghesi et al. 2016).

Monitoring lower-tier species in trophic guilds, such as insectivorous birds, has shown that they are also valuable when used as sentinels to determine pollutant contamination. (Brum et al. 2020). Furthermore, the fact that some species of insects have good resilience to the presence of non-essential metals (Kagata and Ohgushi 2011), those present in sugarcane crops and in the fragments surrounding them, can connect the two environments (Turchin et al. 1991). However, environments that face rapid human-induced change reduce the chances of survival and reproduction of organisms (Sih 2013; Demeyrier and Lambrechts 2016; Hale et al. 2016) and can be defined as ecological or evolutionary traps (Schlaepfer et al. 2002; Gilroy and Sutherland 2007; Robertson et al. 2013; Hale et al. 2015).

We hypothesize that forest birds that inhabit fragments surrounded by sugarcane fields and pastures present some level of aluminum (Al) contamination and may be living in an ecological trap. This study seeks to evaluate the body condition and identify, through feathers, whether wild birds in the understory are contaminated by aluminum in forest fragments surrounded by sugarcane crops and agricultural pastures, characterizing the contaminated spaces as ecological traps for the populations of these fragments, especially those belonging to the insectivorous guild.

## Methods and Materials

### Study Area

**Fig. 1:**
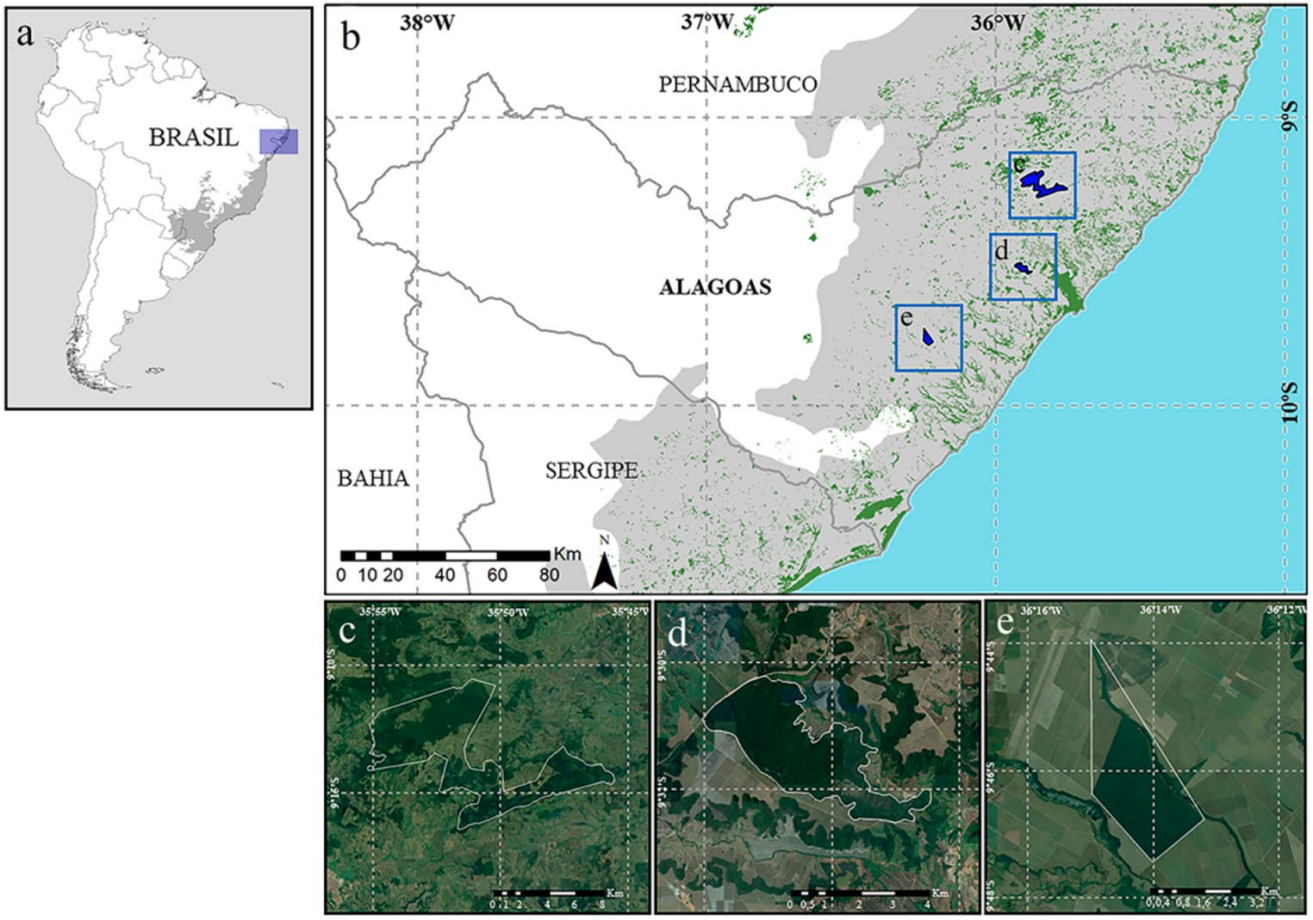
Location of the studied fragments inserted in the Pernambuco Center of Endemism. **C** Murici Ecological Station; **D** Private Natural Heritage Reserve Mata do Cedro; **E** Private Natural Heritage Reserve Dubinha Guimarães

The study was carried out in three fragments of Atlantic Forest protected from the Pernambuco Center of Endemism (Fig. 1) in Alagoas, two surrounded by sugarcane crops and one immersed in agricultural pastures. The Murici Ecological Station (here called Murici) is a federal protected area of restricted use created in 2001 and has an area of approximately 6,116 hectares distributed between the cities of Murici, Flexeiras and Messias, located 50 km from the capital of Alagoas. This region is home to important remnants of the Atlantic Forest interspersed with extensive agricultural systems. One of the most representative sites of Murici, the Fazenda Bananeiras forest, with 2,131 ha, is among the five largest remnants of the Pernambuco Center of Endemism and has a record of many endemic and threatened species (Myers et al. 2000). The Private Natural Heritage Reserve Mata do Cedro (here called Cedro) is a private protected area with permitted use, located in Rio Largo, a municipality neighboring Maceió. It has an area of approximately 500 ha and is surrounded by a matrix of sugar cane. The Private Natural Heritage Reserve Dubinha Guimarães (here called Dubinha) has approximately 690 ha, is also a private protected area with permitted use and is in the municipality of Campo Alegre. It is considered the most extensive area of Atlantic Forest in the state of Alagoas (Lobo-Araújo et al. 2013).

### Sampling

Sampling was carried out in three-day expeditions with 20 h of sampling effort between September 2021 and October 2022. The months were selected considering the calendar of the 2021/2022 sugarcane harvest, whose soil preparation period accompanied the rainfall, until August, and the harvest ended in October 2022. The sampling was based on the random collection of species using 10 lines of ornithological nets installed 300 m from the edge of each fragment, measuring 12 m each. After capture, tarsus length measurements were collected using a digital caliper (0.01 mm) and body mass using dynamometers (10, 30 or 300 g) to calculate the Body Condition Index (BCI).

In addition, 10 feathers were removed from the bird's breast to avoid damaging its flight feathers. Then, the feathers were placed in individual plastic bags and subsequently stored for analysis. The birds received a metal ring (supplied by CEMAVE/IBAMA), for identification and individualization of the collected feather samples. Collections were authorized by the University Ethics Council for the Use of Animals (CEUA Process No. 80/2018) and by the Biodiversity Authorization and Information System (SISBIO—Process No. 23205–12).

### Analytics

Only species that had at least two individuals in each area were used to calculate the BCI. The values were obtained through the residuals of a simple Linear Regression (RL; Peig and Green 2010) between the values of mass (response variable) and tarsus length (explanatory variable) for each species separately, based on the variation observed for the species in the data set (Schulte-Hostedde et al. 2005). Indices with values that are further away from values close to zero (species mean) indicate better (for positive values) or worse (negative values) body condition, when compared with the other individuals (Schulte-Hostedde et al. 2005).

The feathers samples were freeze-dried to remove water through sublimation, weighed, macerated for pulverization, and stored in a desiccator to avoid moisture absorption. Due to very limited sample mass, feathers from individuals of different species collected at the same location and date were analyzed in groups, forming composite samples in order to have triplicates and guarantee the Q/A in the statistics analysis (Table 1). Subsequently, they were digested in a microwave oven with a cavity to mineralize the samples, eliminate organic matter and make them compatible with the measurement technique based on the parameters stablished by the Environmental Protection Agency (EPA). Briefly, 0.0250 ± 0.0001 g of each composite sample was measured and transfered to PTFE flasks followed by the addition of 5.0 mL of HNO_3_ 7 mol L^−1^, 3.0 mL of H_2_O_2_ 30% v v^−1^. The digestion program was performed as following: 3 min at a power of 300 W; 1 min at 0 W; 4 min at 500 W; 3 min at 650 W; 3 min at 1000 W and 15 min of ventilation. The maximum temperature reached during digestion was 185 ºC.

**Table 1:** Mass and BCI values of the analyzed birds and assembly of samples for spectrometric analysis, gathering feathers of species by location and date and concentrations of aluminum (AL) found in feathers of understory forest birds in fragments of the Atlantic Forest of the Pernambuco Center of Endemism in Alagoas, Brazil

Local	Sample	Date	Taxon	Al (mg/kg)	Mass (g)	BCI
Murici	Murici I	10/09/21	*Pyriglena pernambucensis*	257,56	32,4	2,067
			*Chiroxiphia pareola*		18,0	0,462
			*Dendrocincla taunayi*		35,1	–
			*Xiphorhynchus atlanticus*		26,3	− 0,857
			*Platyrinchus mystaceus niveigularis*		14,0	1,556
	Murici II	10/09/21	*Schiffornis turdina*	265,51	42,6	3,863
			*Schiffornis turdina*		36,4	− 2,287
			*Schiffornis turdina*		38,6	0,129
Cedro	Cedro III	09/10/22	*Schiffornis turdina*	57,92	35,0	− 4,619
			*Schiffornis turdina*		36,0	− 2,785
	Cedro IV	09/02/22	*Ceratopipra rubrocapilla*	273,58	2,5	− 6,928
			*Ceratopipra rubrocapilla*		13,0	− 2,249
			*Ceratopipra rubrocapilla*		5,0	− 4,571
			*Ceratopipra rubrocapilla*		15,5	5,255
	Cedro V	09/02/22	*Myrmotherula axillaris*	381,34	9,0	− 0,854
			*Picumnus exilis*		9,0	–
			*Basileuterus culicivorus*		10,0	1,765
			*Conopohaga melanops*		24,0	0,894
			*Xenops minutus alagoanus*		10,0	0,507
Dubinha	Dubinha VI	28/08/21	*Chiroxiphia pareola*	552,62	4,0	− 0,385
			*Veniliornis affinis*		14,0	
			*Arremon taciturnus*		5,0	3,608
	Dubinha VII	03/10/21	*Chiroxiphia pareola*	323,73	20,5	19,0
			*Chiroxiphia pareola*		–	–
			*Xenops minutus alagoanus*		14,9	9,0
			*Hemitriccus griseipectus*		16,9	9,0
			*Thamnophilus aethiops distans*		24,2	24,0
			*Thamnophilus aethiops distans*		21,0	23,0
			*Arremon taciturnus*		25,8	27,0
	Dubinha VIII	13/02/22	*Chiroxiphia pareola*	344,31	20,0	3,829
			*Thamnophilus aethiops distans*		25,0	4,805
			*Myrmotherula axillaris*		7,0	− 0,976
			*Ceratopipra rubrocapilla*		16,0	6,637
			*Arremon taciturnus*		25,0	6,937
			*Hemitriccus mirandae*		10,0	–

After digestion, the digested samples were quantitatively transferred to polyethylene flasks wich were filled up to a final volume of 30 mL with deionized water. Analytical blanks were prepared as well as the samples for monitoring of analytical quality control. Then, inorganic analytes were determined by inductively coupled plasma optical emission spectrometry (ICP OES) using a spectrometer Agilent Technologies Model 720 series (Mulgrave, Australia). The applied conditions were: cyclonic nebulizer chamber (Single Pass), OneNeb nebulizer, radiofrequency power of 1.2 kW, plasma gas flow of 15.0 L min^−1^, auxiliary gas flow of 1.5 L min^−1^, nebulizer gas flow rate of 0.75 L min^−1^ and wavelength of the atomic line was 396.152 nm. The calibration method was external one and the calibration curve was obtained with standard solutions of the aluminum in the range of 0.1—4.0 mg L^−1^. The limits of detection and quantification were, rescpectively, 4.54 and 15 mg kg^−1^.

Soil analysis of sugarcane fields around the Mata do Cedro and Dubinha Private Natural Reserves (RPPN) was provided by the owning companies (Usina Utinga Leão S/A and Industrial Porto Rico S/A, respectively). Furthermore, soil analysis of agricultural pastures around the Murici ESEC was obtained by the Center for Agricultural Sciences of the Federal University of Alagoas.

## Results

When evaluating the distribution of BCI values for bird communities in different fragments (Table 1), a higher prevalence of individuals with lower BCI body condition was observed in Cedro, mainly in *Schiffornis turdina* (− 4,619) and *Ceratopipra rubrocapilla* (− 6,928). In Murici, *Xiphorhynchus atlanticus*, a nationally threatened species, presented a negative BCI value (-0,857) indicating low body condition (Table 1).

The feathers of 35 birds belonging to 15 species were divided into eight samples. The samples and n value per species per site is detailed in Table 1. The presence of aluminum was detected in all of them (Table 1). In the Murici II (265,51 mg/kg) and Cedro III (57,92 mg/kg) samples containing only *Schiffornis turdina* feathers, it was verified that the birds from Murici a much higher aluminum concentration birds from Cedro (Table 1). On the other hand, samples from Cedro (273,58 mg/kg) e Dubinha (552,62 mg/kg) composed of feathers from various species on different dates, had higher values than the Murici samples (257,56 mg/kg). The Dubinha VI sample combining feathers from *Arremon taciturnus*, *Venilliornis affins* and *Chiroxiphia pareola* obtained the highest concentration of aluminum (552.62 mg/kg), while the Murici I sample containing feathers from *Pyriglena pernambucensis*, *Chiroxiphia pareola*, *Xiphorhynchus atlanticus*, *Platyrinchus mystaceus niveigularis* and *Dendrocincla taunayi* had the lowest aluminum contamination value (257,56 mg/kg) (Table 1).

Soil analysis around the studied areas show Aluminum concentrations between 0.11 and 1.0 meq/100 ml (Supplementar Material, Tables 1, 2 3), with the highest concentrations (mean) found in the soil around Murici (0.66 ± 0.29) (Supplementar Material, Table 4).

## Discussion

This study assessed body condition and points to the presence of aluminum in samples of feathers from understory forest birds in fragments of the Atlantic Forest of the Pernambuco Endemism Center in Alagoas, Brazil. In fact, Aluminum is the third most common element in the Earth's crust, so it is certainly no surprise that this element was detected in feathers. Nonetheless, the presence of trace elements such as aluminum in feathers can have potential negative effects on bird health. For instance, elements like cadmium and barium, which are often found alongside aluminum, have been shown to inversely correlate with body mass and wing length, suggesting detrimental impacts on the corporal condition of birds (Bada and Omotoriogun 2019).

Birds of the species *S. turdina* from Murici had higher aluminum concentrations than birds of the same species from Cedro. On the other hand, individuals from Cedro presented lower body condition. In fact, there is a difference between the environmental matrix that surrounds Murici, composed mainly of pastures, and Cedro, surrounded by sugar cane plantations. Both matrices have different fertilization cycles and use different chemical compounds in their crops, which may explain the difference found between aluminum concentrations in *S. turdina* in the two areas. Furthermore, the larger size, the smaller edge effect, as well as the better conservation status of Murici can offer better food resources that ensure the best body condition of its avifauna.

Concentrations of about 200 mg/kg can be considered as normal levels for aluminum in bird feathers (Shafaeipour et al. 2024). Our study, very close to this value were observed in most of the sampled areas. However, it is worth noting the higher concentration in Murici, which can be explained by the lower pH (4.91, Table 4), characterizing an acidic soil. The ESEC Murici, even without having sugarcane crops in its surroundings, presented levels of aluminum contamination considered high, proving to be a threat to the local fauna. Indeed, in more acidic soils, aluminum in the form of Al^3^⁺ ions becomes more soluble and biologically available, increasing its concentration in forest waters and soils (Wesselink 1990; Cronan and Schofield 1990; Sparling and Lowe 1996; Jiang et al. 2018).

Thus, available aluminum can accumulate in tissues and contaminate forest birds, mainly indirectly, via the food chain and prey reduction (Sparling and Lowe 1996). This reduction of invertebrates as a food source can negatively impact the body condition of forest birds by limiting the availability of essential nutrients. All of this can lead to decreased reproductive success, growth, and survival of birds (Rosseland et al. 1990; Sparling and Lowe 1996). Previous studies observed that aluminum toxicity was responsible for the reduction in the brood of *Parus atricapillus* and *Dendroica pensylvanica* and their change in foraging behavior (Carriere et al. 1986; Sparling 1990; Scheuhammer 1991).

It is important to highlight that most of the species live in the lower substrates of the forests, feeding on insects, making them easy targets for contamination by aluminum and other possible contaminants found in these fragments. Therefore, the consumption of insects by birds, such as *S. turdina*, may increase their exposure to aluminum contamination. In addition, contamination in *X. atlanticus* samples demonstrates the need for attention for the species and other insectivores in one of the regions with the most threatened birdlife on the planet (Collar et al. 1992; Brooks and Balmford 1996; Lees and Pimm 2015).

Although direct studies with forest birds are scarce, research with urban birds exposed to toxic metals, including aluminum, shows adverse effects on immunological, antioxidant, and hematological parameters, even when apparent body condition is not immediately affected (Scheuhammer 1987; Li et al. 2021). Therefore, we emphasize the importance of monitoring endemic and threatened species that still survive in small fragments such as the Dubinha Private Natural Park. Taxa such as *Xenops minutus alagoanus*, *Thamnophilus aethiops distans*, and *Hemitriccus mirandae*, despite presenting positive body condition indices, may have their populations compromised in the medium to long term.

## Conclusion

Our study demonstrated the presence of aluminum in the feathers of several threatened and endemic bird species from the Pernambuco Center of Endemism, a region of the Brazilian Atlantic Forest that concentrates the most threatened avifauna on the planet.

Since exposure to this metal can affect the bird’s physiology, behavior, and resistance to disease, the poor body condition observed in some of the analyzed individuals may compromise reproductive success and population recovery of the most sensitive species, which tend to have already reduced populations for other reasons.

Finally, the fact that these birds continue to live in a location that diminishes their survival and reproduction corroborates our ecological trap hypothesis and serves as a warning about the need for attention to the protected areas studied in the face of the threat of local extinction.

## Supplementary Information

Below is the link to the electronic supplementary material.

Supplementary file1 (DOCX 22 kb)
