# Supplementary material for: Aluminium Exposure in Understory Birds of Atlantic Forest Fragments in Brazil
Source: Bull Environ Contam Toxicol. 2026 Apr 17;116(5):90. doi: 10.1007/s00128-026-04239-6 (PMC13090288; doi:10.1007/s00128-026-04239-6)
Supplement: Supplementary file 1 — Supplementary file1 (DOCX 22 kb) [file 128_2026_4239_MOESM1_ESM.docx]

**Supplementary Material**

**Table 1**. Soil analysis of sugarcane crops around the RPPN Dubinha Guimarães. Source: Porto Rico Industry

| Certificate Nº: 043951 |
| --- |
| Origin: Industrial Porto Rico |
| Groups: soil |
| March 2023. |

|  |  |  |  |  |  |  |  |  |  |  |
| --- | --- | --- | --- | --- | --- | --- | --- | --- | --- | --- |
|  | **Samples** | | | | | | | | | |
| **Parameters** | 94164 | 94165 | 94166 | 94167 | 94168 | 94169 | 94170 | 94171 | 94172 | 94173 |
| pH (in water) | 5,3 | 6,5 | 6,1 | 6,3 | 6,0 | 6,5 | 5,2 | 6,8 | 6,3 | 6,3 |
| Na (ppm) | 9 | 9 | 16 | 9 | 16 | 10 | 18 | 14 | 42 | 5 |
| P (ppm) | 10 | 8 | 9 | 19 | 7 | 7 | 7 | 28 | 26 | 6 |
| K (ppm) | 21 | 21 | 41 | 21 | 43 | 23 | 34 | 29 | 111 | 35 |
| Ca+Mg (meq/100ml) | 3,5 | 1,4 | 4,6 | 1,8 | 4,6 | 3,0 | 5,6 | 0,9 | 6,4 | 3,0 |
| Ca (meq/100ml) | 1,9 | 0,8 | 4,2 | 1,1 | 2,5 | 1,8 | 3,4 | 0,7 | 3,8 | 1,6 |
| Mg (meq/100ml) | 1,6 | 0,6 | 0,4 | 0,7 | 2,1 | 1,2 | 2,2 | 0,2 | 2,6 | 1,4 |
| Al (meq/100ml) | 0,00 | 0,35 | 0,00 | 0,00 | 0,00 | 0,00 | 0,10 | 0,93 | 0,00 | 0,00 |
| H+Al (meq/100ml) | 4,0 | 3,5 | 1,3 | 2,4 | 2,6 | 4,6 | 3,6 | 7,6 | 0,4 | 3,3 |

**Table 2**. Soil analysis of sugarcane crops around the RPPN Mata do Cedro. Source: Utinga Leão Plant.

| Certificate Nº: 040112 |
| --- |
| Origin: Usina Utinga Leão |
| Groups: soil |
| May 2022. |

| **Samples** | | | | | | | | | |  |
| --- | --- | --- | --- | --- | --- | --- | --- | --- | --- | --- |
| **Parameters** | 432 | 433 | 434 | 435 | 436 | 437 | 438 | 439 | 440 | 441 |
| pH (in water) | 4,9 | 4,9 | 5,5 | 5,7 | 6,4 | 6,2 | 5,2 | 5,3 | 5,7 | 5,6 |
| Na (ppm) | 12 | 11 | 22 | 28 | 12 | 0 | 12 | 8 | 10 | 9 |
| P (ppm) | 54 | 41 | 41 | 15 | 83 | 48 | 0 | 7 | 10 | 7 |
| K (ppm) | 68 | 27 | 20 | 13 | 26 | 17 | 32 | 18 | 59 | 30 |
| Ca+Mg (meq/100ml) | 2,9 | 2,8 | 4,1 | 3,8 | 6,3 | 5,1 | 3,1 | 3,5 | 4,5 | 4,1 |
| Ca (meq/100ml) | 1,4 | 1,4 | 2,0 | 1,9 | 3,2 | 2,6 | 1,5 | 1,7 | 2,2 | 2,1 |
| Mg (meq/100ml) | 1,5 | 1,4 | 2,1 | 1,9 | 3,1 | 2,5 | 1,5 | 1,8 | 2,3 | 2,0 |
| Al (meq/100ml) | 0,69 | 0,81 | 0,46 | 0,45 | 0,00 | 0,00 | 0,40 | 0,35 | 0,11 | 0,17 |
| H+Al (meq/100ml) | 6,3 | 5,6 | 3,5 | 3,1 | 2,6 | 3,3 | 5,3 | 4,8 | 4,8 | 4,2 |

**Table 3**. Soil analysis of agricultural pastures around ESEC-Murici. Source: UFAL.

| Certificate Nº:046261 |
| --- |
| Origin: FUNDEPES-UFAL |
| Groups: soil |
| August 2023 |

| **Samples** | | | | | | | | | | | |  |
| --- | --- | --- | --- | --- | --- | --- | --- | --- | --- | --- | --- | --- |
| **Parameters** | 248 | 249 | 250 | 251 | 252 | 253 | 254 | 255 | 2576 | 257 | 258 | 259 |
| pH (in water) | 5,1 | 5,2 | 4,7 | 4,8 | 4,7 | 4,7 | 5,3 | 5,1 | 4,8 | 4,9 | 4,9 | 4,7 |
| Na (ppm) | 20 | 19 | 19 | 19 | 19 | 19 | 20 | 18 | 19 | 18 | 18 | 18 |
| P (ppm) | 69 | 23 | 59 | 41 | 28 | 18 | 119 | 5 | 36 | 10 | 8 | 4 |
| K (ppm) | 43 | 38 | 23 | 22 | 24 | 23 | 47 | 32 | 19 | 17 | 19 | 19 |
| Ca+Mg (meq/100ml) | 3,6 | 2,2 | 1,2 | 1,6 | 3,1 | 2,0 | 4,8 | 2,7 | 1,8 | 2,8 | 1,9 | 1,3 |
| Ca (meq/100ml) | 2,4 | 1,9 | 0,9 | 1,3 | 2,7 | 1,3 | 3,8 | 1,8 | 1,1 | 1,8 | 1,7 | 1,0 |
| Mg (meq/100ml) | 1,2 | 0,3 | 0,3 | 0,3 | 0,4 | 0,7 | 1,0 | 0,9 | 0,7 | 1,0 | 0,2 | 0,3 |
| Al (meq/100ml) | 0,77 | 0,52 | 0,92 | 0,81 | 0,98 | 1,0 | 0,18 | 0,15 | 0,63 | 0,54 | 0,56 | 0,90 |
| H+Al (meq/100ml) | 8,5 | 6,8 | 6,0 | 5,5 | 8,0 | 7,5 | 7,2 | 4,3 | 4,2 | 3,5 | 0,57 | 0,90 |

**Table 4.** Aluminum (meq/100ml) and pH values ​​(in water) measured in the soil surrounding the studied areas.

|  | **Aluminum** | | | | | | | | | | | | **Mean + SD** |
| --- | --- | --- | --- | --- | --- | --- | --- | --- | --- | --- | --- | --- | --- |
|  | Lot 1 | Lot 2 | Lot 3 | Lot 4 | Lot 5 | Lot 6 | Lot 7 | Lot 8 | Lot 9 | Lot 10 | Lot 11 | Lot 12 |  |
| Dubinha | 0 | 0,35 | 0 | 0 | 0 | 0 | 0,1 | 0,93 | 0 | 0 |  |  | 0,14±0,51 |
| Cedro | 0,69 | 0,81 | 0,46 | 0,45 | 0 | 0 | 0,4 | 0,35 | 0,11 | 0,17 |  |  | 0,34±0,28 |
| Murici | 0,77 | 0,52 | 0,92 | 0,81 | 0,98 | 1 | 0,18 | 0,15 | 0,63 | 0,54 | 0,56 | 0,9 | **0,66±0,29** |
|  | Ph | | | | | | | | | | | |  |
| Dubinha | 5,3 | 6,5 | 6,1 | 6,3 | 6 | 6,5 | 5,2 | 6,8 | 6,3 | 6,3 |  |  | 6,13±0,51 |
| Cedro | 4,9 | 4,9 | 5,5 | 5,7 | 6,4 | 6,2 | 5,2 | 5,3 | 5,7 | 5,6 |  |  | 5,54±0,50 |
| Murici | 5,1 | 5,2 | 4,7 | 4,8 | 4,7 | 4,7 | 5,3 | 5,1 | 4,8 | 4,9 | 4,9 | 4,7 | **4,91±0,22** |
